# Unveiling *Prasinovirus* diversity and host specificity through targeted enrichment in the South China Sea

**DOI:** 10.1093/ismeco/ycae109

**Published:** 2024-08-29

**Authors:** Julie Thomy, Frederic Sanchez, Camille Prioux, Sheree Yau, Yangbing Xu, Julian Mak, Ruixian Sun, Gwenael Piganeau, Charmaine C M Yung

**Affiliations:** Sorbonne Université, CNRS, Laboratoire de Biodiversité et Biotechnologies Microbiennes (LBBM), Observatoire Océanologique, F-66650 Banyuls/Mer, France; Department of Oceanography, School of Ocean and Earth Science and Technology (SOEST), University of Hawai‘i at Mānoa, Honolulu, HI 96822, United States; Sorbonne Université, CNRS, Biologie Intégrative des Organismes Marins (BIOM), UMR 7232, Observatoire Océanologique, F-66650 Banyuls/Mer, France; Sorbonne Université, CNRS, Laboratoire de Biodiversité et Biotechnologies Microbiennes (LBBM), Observatoire Océanologique, F-66650 Banyuls/Mer, France; Centre Scientifique de Monaco, 8 Quai Antoine 1er, Monaco, MC 98000, Principality of Monaco; Sorbonne Université, CNRS, Laboratoire de Biodiversité et Biotechnologies Microbiennes (LBBM), Observatoire Océanologique, F-66650 Banyuls/Mer, France; Department of Ocean Science, The Hong Kong University of Science and Technology, Hong Kong SAR, China; Department of Ocean Science, The Hong Kong University of Science and Technology, Hong Kong SAR, China; Department of Ocean Science, The Hong Kong University of Science and Technology, Hong Kong SAR, China; Sorbonne Université, CNRS, Laboratoire de Biodiversité et Biotechnologies Microbiennes (LBBM), Observatoire Océanologique, F-66650 Banyuls/Mer, France; Southern Marine Science and Engineering Guangdong Laboratory (Guangzhou), The Hong Kong University of Science and Technology, Hong Kong SAR, China; Department of Ocean Science, The Hong Kong University of Science and Technology, Hong Kong SAR, China; Southern Marine Science and Engineering Guangdong Laboratory (Guangzhou), The Hong Kong University of Science and Technology, Hong Kong SAR, China

**Keywords:** Nucleocytoviricota, Mamiellophyceae, Mamiellales, host range, Prasinovirus, diversifying selection, horizontal gene transfer

## Abstract

Unicellular green picophytoplankton from the Mamiellales order are pervasive in marine ecosystems and susceptible to infections by prasinoviruses, large double-stranded DNA viruses within the *Nucleocytoviricota* phylum. We developed a double-stranded DNA virus enrichment and shotgun sequencing method, and successfully assembled 80 prasinovirus genomes from 43 samples in the South China Sea. Our research delivered the first direct estimation of 94% accuracy in correlating genome similarity to host range. Stirkingly, our analyses uncovered unexpected host-switching across diverse algal lineages, challenging the existing paradigms of host–virus co-speciation and revealing the dynamic nature of viral evolution. We also detected six instances of horizontal gene transfer between prasinoviruses and their hosts, including a novel alternative oxidase. Additionally, diversifying selection on a major capsid protein suggests an ongoing co-evolutionary arms race. These insights not only expand our understanding of prasinovirus genomic diversity but also highlight the intricate evolutionary mechanisms driving their ecological success and shaping broader virus–host interactions in marine environments.

## Introduction

Over the past five decades, the discovery of diverse viruses in freshwater and marine algae has unveiled their pervasive presence and ecological importance [1, 2] . These include viruses infecting cosmopolitan unicellular photosynthetic eukaryotes, such as the coccolithophore *Gephyrocapsa huxleyi* [3–5], haptophytes including *Phaeocystis* spp. [2, 6], and picoeukaryotes like *Micromonas* [7]. Many of these viruses, belonging to the *Nucleocytoviricota* phylum [8], are characterized by large double-stranded DNA genomes that can be up to 2.7 Mbp [9, 10]. These giant viruses profoundly impact phytoplankton population dynamics [11, 12] and are positively correlated with carbon export in marine ecosystems, highlighting the ecological importance of phytoplankton–virus interactions in the carbon cycle [13].

Advances in metagenomic sequencing and single-virus genomics have dramatically enhanced our knowledge of marine viral diversity and distribution [14, 15]. International sequencing efforts have identified over 2500 giant virus metagenome-assembled genomes (GVMAGs) from marine environments [16, 17]. Furthermore, single-virus genomics, which involves sequencing individual virus-like particles sorted by flow cytometry to obtain single-virus assembled genomes (vSAGs), has deepened our insights into marine viral genomic diversity [18]. However, the specific hosts and host ranges of these GVMAGs and vSAGs remain largely unknown, often inferred only from cultured strains [16, 19, 20].

To address these limitations, we developed a host-targeted virus enrichment strategy that enables detailed investigation of viral genome diversity and direct linkage to specific host species. Our research has focused on the picophytoplankton order Mamiellales, an ideal model for this approach due to their high abundance and cosmopolitan distribution [21]. These algae, diverging from a common ancestor between 330 and 640 million years ago [21], are common hosts for the widespread prasinoviruses [15, 16, 22]. Their abundance in marine environments allows for direct virus isolation without the need for a concentration step. We applied this strategy in the South China Sea (SCS), an underexplored region for phytoplankton research [23]. As a major sea in the northwestern Pacific Ocean, SCS is crucial for the water mass exchange between the Pacific and Indian Oceans. Our approach successfully identified multiple prasinovirus strains in the natural environment and linked them to their algal hosts. This study provides valuable insights into the dynamic interplay between viruses and their Mamiellales hosts, revealing genetic variations, evolutionary relationships, and mechanisms such as horizontal gene transfer that drive their evolution.

## Materials and methods

### Water sampling

**Figure 1:**
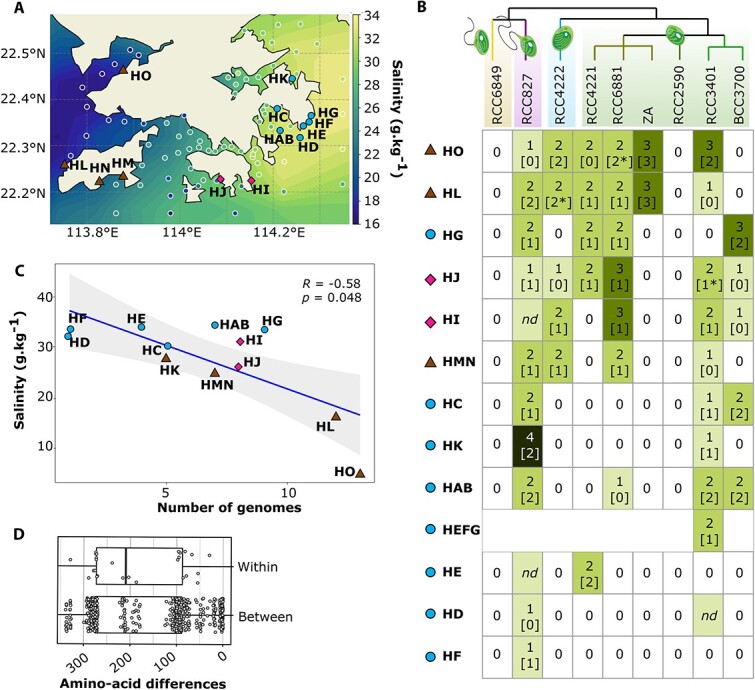
Sampling seawater characteristics and number of virus assemblies recovered. (A) The spatially interpolated monthly averaged sea surface salinity in June 2020 from the observational data assimilating from Global Ocean forecasting system 3.1 (#expt93.0, from the 41-layer HYCOM + NCODA global 1/12° analysis). Observational data from the Hong Kong environmental protection department averaged over the same period are represented by white circles with shading. The water sampling locations reported in this study are indicated by triangles, circles, and diamonds, with individual samples labeled with two letters (e.g. HA to HO), and pooled samples indicated by multiple letters (e.g. HEFG results from pooling HE, HF, and HG). (B) Environmental water-challenged culture matrix representing lysis or no lysis of 117 Mamiellales cell cultures. The dendrogram illustrates the phylogenetic relationships among the Mamiellales strains. The genera are *Mantoniella*, *Micromonas*, *Bathycoccus*, and *Ostreococcus*. Branch colors indicate three distinct *Ostreococcus* species: *O. tauri*, *O. mediterraneus*, and *O. lucimarinus*. The matrix is ordered by the frequency of successful lyses along the vertical axis. The total number of virus assemblies recovered in each sampling site is indicated within the boxes, with the number of virus assemblies kept for in-depth analyses in brackets. Asterisk (*) denotes the presence of two distinct DNA *polB* genes detected in the same genome. (C) Linear correlation between the number of viral genome assemblies and salinity. The shaded area represents the 95% confidence interval. *R*, Spearman correlation coefficient; *P*, *P*-value. (D) Boxplot showing the number of amino acid differences from all pairwise viral *polB* gene alignments between lysates and from pairwise *polB* gene alignments from sequences within the same lysate.

Seawater samples were collected from 13 coastal habitats around Hong Kong from February to May and September to November 2020 (Fig. 1A). Eastern sites (HAB-HK) are influenced by shelf and oceanic waters from the SCS, while western sites (HM-HO) are influenced by the Pearl River freshwater discharge. Duplicate 50-ml samples of surface seawater were collected using polycarbonate bottles. Temperature, dissolved oxygen, salinity, pH, and turbidity were measured with a YSI ProDSS meter. Samples were syringe filtered through 0.45-μm polyethersulfone filters (PES) and stored at 4°C.

### Eukaryotic phytoplankton culturing

The eukaryotic phytoplankton strains used as potential hosts for isolating viruses included *Bathycocccus prasinos* (RCC4222), *Mantonellia* sp. (RCC6849), *Micromonas commoda* (RCC827), and *Ostreococcus* sp. (RCC4221, RCC2590, RCC3401, RCC6881, BCC37000, and ZA5.1). All strains were cultivated in L1 medium (NCMA) made with autoclaved seawater (MOLA station: 42°27′11″ N, 3°8′42″ E), diluted to a salinity of 30 g l^−1^, and filter-sterilized through 0.22-μm filters. Cultures were maintained under a 12 h:12 h light/dark (50 μmol m^−2^ s^−1^ white light) at 20°C. To verify species identity, we amplified the full eukaryotic *18S rDNA* gene of the algal strains at the start and end of the experiment using established primers and performed Sanger sequencing [24].

### Virus enrichment

Five milliliters of each of the thirteen 0.45-μm-filtered seawater samples was added to 10 ml of nine different microalgal cultures in culture flasks and monitored color changes daily against a control for up to 8 days under the previously described culturing conditions. Upon observing discoloration, the lytic property was confirmed by re-inoculating 5 ml of the 0.45-μm-filtered lysate into 10 ml of fresh microalgae culture. After confirming discoloration, the volume of each of the 52 lysates was gradually doubled using fresh culture until reaching 145 ml. Each lysate was then filtered through a 0.45-μm PES filter to remove debris and bacteria. Virus-like particles (VLPs) concentrations were estimated using a Beckman-Coulter Cytoflex flow cytometer [25]. Lysates from geographically close samples and the same strain with <5 × 10^5^ VLPs ml^−1^ were pooled, reducing the number of lysates from 52 to 44. The experimental pipeline is synthesized in Supplementary Fig. 1. All statistical analyses were performed with R (v4.3.2) [26].

### DNA extraction and sequencing

Viral particles from the 44 lysates were concentrated using a 0.1-μm-pore-size polycarbonate filter (Millipore; VCTP0470) and incubated in CTAB lysis buffer (2% CTAB, 100 mM Tris–HCl [pH = 8], 20 mM EDTA, 1.4 M NaCl, and 0.2%, 10 mM DTT and 0.1 mg ml^−1^ proteinase K) at 60°C for 1 to 2 h, following a published extraction protocol [27]. DNA quality was evaluated by a Nanodrop at 260/230 nm and 260/280 nm, and confirmed by 0.8% agarose gel electrophoresis. DNA quantification was performed using the Quantus fluorimeter with the QuantiFluor dsDNA system kit. Due to one extraction failure (RCC827 at the HI site), 43 short-insert paired-end libraries (2 × 150 bp) were sequenced using the Illumina Nextseq550 system at the Bioenvironment platform of the University of Perpignan.

### Construction of prasinovirus genomes

Between 308- and 582-Mb sequences were obtained per lysate, totaling 37 million bp across 43 lysates. Each sample was assembled with metaSPAdes (version 3.15.1) (−k 55,77,99 127 —meta) [28], yielding 47 469 contigs >1 kbp. Supervised binning of the contigs into distinct virus genomes was conducted based on tetranucleotide frequency and coverage within each lysate using Anvi’o v7.1 [29]. In the sample OtV-6881-HJ, no viral sequences were initially identified due to dominant bacterial signals, but subsequent use of VirSorter [30] with default settings enabled the identification of one viral genome. In total, 80 viral genomes of at least 100 kb each were reconstructed (Supplementary Table 1), which corresponds to $\sim$50% of known prasinovirus genome length. For genomes with 2 to 15 contigs showing overlap, manual scaffolding into a single large contig was conducted using Geneious (v11.0.3 + 7). The genomes were then reoriented based on their alignment with the most closely related viral reference genome using Mummer (v4.0.0beta2) [31]. Assemblies with >15 contigs were retained without manual scaffolding.

To identify highly similar viral sequences among samples from neighboring locations, whole-genome Average Nucleotide Identity (wgANI) analysis was conducted using skani [32]. A conservative threshold of wgANI >98% (corresponding to ANI >99% and aligned fraction >99%) was applied to cluster highly similar viral sequences. This threshold balances the need to distinguish meaningful genomic variation against potential sequencing errors and other technical artifacts. A dereplication step was applied to retain only one representative genome from each cluster. Genome completeness was further assessed based on the presence of at least three out of six *Nucleocytoviricota* marker genes (SFII, PolB, TFIIB, TopoII, A32, VLTF3) using the script “ncldv_markersearch” [17].

### Genome annotation

Protein-coding genes were annotated using Prokka (v1.14.5) [33] with the following parameters: *e*-value of 1*e*^−5^, and genetic code standard (—gcode 1), specifying “virus” as the taxonomic kingdom and applying “metagenome.” Further functional annotations were performed using BLASTp against the RefSeq protein database using Diamond (v2.0.8) [34] with an *e*-value cutoff of 1*e*^−5^, supplemented by annotations from the EggNOG-mapper toolkit [35] and the InterProScan database (v5.44-79.0) [36] with default settings. Transfer RNA were predicted using tRNAscan-SE (v2.0.2) [37] with default settings.

### Phylogenetic analysis

Maximum likelihood (ML) concatenated phylogenetic trees were reconstructed using 6 *Nucleocytoviricota* marker genes [17] from 51 viral genomes and 22 reference prasinovirus genomes, with the chlorovirus PBCV-1 genome as an outgroup. Protein sequences were aligned using the L-INS-i method in MAFFT [38] (v7.313). Alignments were refined by removing positions with >50% gaps using Goalign (v0.3.2) [39] and manually inspected. ML phylogenetic analysis of single and concatenated proteins were conducted using IQ-TREE (v2.0.6) [40], based on 3360 amino acid positions (Supplementary Figs. 2–7). The best-fitting model, LG + F + R4, was selected using the fast model-selection (−m MFP) [41] based on the Bayesian information criterion (BIC). Branch support were evaluated using 1000 Shimodaira–Hasegawa-like approximation likelihood ratio test and 1000 ultra-fast bootstrap approximations [42]. The phylogenetic trees were visualized with the Interactive Tree Of Life (iTOL) v6 [43].

### Comparative genomics analysis

Orthogroups among 51 viral genomes from this study and 22 reference prasinovirus genomes were identified using OrthoFinder (v2.5.4) [44] with default settings. Hierarchical clustering was performed based on the Euclidean distance of the presence/absence orthogroups patterns, with the “ward.D2” linkage method. Statistical significance was assessed with approximately unbiased *P*-values derived from 1000 bootstrap replicates using the pvclust R package (v2.2-0). Orthogroup distribution across genomes was visualized in a heatmap using the ggplot2 R package (v3.4.0).

Pan-genome analysis was performed using PANGP [45] to determined core and total genes across 51 studied and 22 reference prasinovirus genomes (Supplementary Table 2). Average gene counts from each iteration were plotted, and the pan-genome curve was fitted using a power-law regression based on Heaps’ law [46]. In this model, *α* values between 0 and 1 indicate an open, infinitely expanding pan-genome as more genomes are added, while values outside this range suggest a closed pan-genome that approaches a plateau.

### Metagenomic analysis

To compare the novelty of SCS viruses with marine viral metagenomes, a BLASTx search was performed using the *polB* protein from SCS viral genomes against the GOEV database. Criteria for relatedness included an *e*-value <1*e*^−5^, protein sequence identity >80%, and alignment length >500 amino acids. *PolB* alignment of 108 sequences (39 GVMAGs, 46 SCS genomes, 22 prasinovirus references, and PBCV-1) was performed using MAFFT (v7.313) with L-INS-I method [38]. Positions with >50% gaps were removed with Goalign (v0.3.2) [39] and manually inspected. A ML phylogenetic tree based on full *polB* was constructed using IQ-TREE (v2.0.6) [40]. The optimal substitution model, Q.insect+I + R5, was selected based on BIC using a fast model-selection method [41]. Branch support was computed using previously described methods. Phylogenetic trees were visualized using iTOL (v6) [43].

### Detection of amino acid sites under diversifying selection

The ratio *ω*, representing non-synonymous (*dN*) to synonymous substitutions (*dS*), elucidates protein evolution effects: *ω* <1 indicates purifying selection, *ω* = 1 suggests neutral evolution, and *ω* >1 suggests positive or diversifying selection. We applied different codon models to the Major Capsid Protein (MCP6) gene: M0 (constant *ω*), M1a (two site classes: *ω*_0_ = 1 and *ω*_1_ < 1), and M2a (three site classes: *ω*_0_ = 1, *ω*_1_ < 1, *ω*_2_ > 1) using PAML4.8 [47]. In cases of *dS* saturation (*dS* > 1) across the dataset, it was split into sub-datasets corresponding to monophyletic groups for separate analyses. The best model was selected based on the highest likelihood and significance in a nested likelihood ratio test (*P* < .05) [47, 48]. Degrees of freedom were 1 for M1a versus M0 and 2 for M2a versus M1a. Three-dimensional structures of proteins with identified selection sites were predicted using AlphaFold v2.0 on the Colab server, with default settings [49], and were visualized using PyMol v3.9 (Schrödinger).

### Horizontal gene transfer analyses

To identify recent horizontal gene transfer (HGT) among the 51 viral genomes and potential hosts, a BLASTp search was performed against the NCBI nr database targeting Mamiellophyceae, with an *e*-value threshold of 1*e*^−5^ and a minimum identity of 60%. This search was extended to include cultivated and uncultivated *Nucleocitoviricota* genomes using the same criteria. For uncultivated genomes, an additional amino acid similarity search was performed against the GOEV database. Genes ubiquitous across all the nucleocytoplasmic large DNA viruses (NCLDVs) were excluded to focus on recent HGT events. Phylogenetic trees were reconstructed using previously described methods.

## Results and discussion

### Dominant and diverse prasinoviruses in the South China Sea

We conducted 117 virus enrichment experiments across 13 distinct water samples from the SCS (Fig. 1A) using nine algal strains, representing four genera and six species (Fig. 1B, Supplementary Fig. 1), and successfully obtained 52 viral lysates. Due to low virus counts detected by flow cytometry, we pooled nearby samples from similar habitats (HMN and HEFG) to increase the probability of successful DNA extraction, ultimately obtaining sequencing reads from 43 lysates. The infection success rate varied significantly among the strains (Fig. 1B) (Chi square, *P*-value < 10^−6^, *df* = 5), with viruses lysing seven out of nine strains. *Micromonas commoda* and *Ostreococcus lucimarinus* consistently produced lysates upon exposure to water from all sites, while *Mantoniella* sp. and *Ostreococcus mediterraneus* yielded none.

DNA sequences from each of the 43 lysates were assembled separately and the assembled contigs were binned into 80 high-contiguity viral genome assemblies (>100 kbp) (Supplementary Table 1), each derived from one of the 43 sequenced lysates. No significant difference was observed in the number of viral genome assemblies among different host strains (one-way analysis of variance, *df* = 6, *P*-value = .42), with an average of 1.9 virus assemblies per lysate. This highlights the widespread occurrence of multiple prasinovirus strains infecting a single algal strain, while no evidence of co-infection was observed in this case. Notably, no non-prasinovirus viral DNA, such as circovirus-like viruses [50], previously reported to potentially infect *Micromonas* [51], was detected. RNA viruses were not targeted in this study; however, the predominance of DNA viruses was clear, with only one lysate failing to yield DNA sequences. The absence of other DNA viruses could be due to their size, either smaller (<100 nm diameter) or larger (>450 nm diameter) than our filtration cutoff, or their low presence in the sampled waters.

Prasinoviruses were ubiquitous in the sampled area, with their distribution significantly influenced by water properties and coastal upwelling dynamics. Specifically, lysates were obtained from a wider range of Mamiellales strains and more prasinovirus genomes from the western sampling stations near the Pearl River Estuary (HO and HL, Fig. 1B), where freshwater runoff impacts local water conditions. In contrast, we obtained fewer lysates and virus genomes from the eastern regions (HE, HF, and HD; Fig. 1B). This distribution pattern aligns with previous studies on phytoplankton communities in the region, particularly the abundance of *Micromonas*, which is influenced by environmental factors, especially salinity [52]. Further analysis showed a significant negative correlation between virus genome counts and salinity (Spearman *ρ* = −0.58, *P*-value = .048, Fig. 1C), but no significant correlations with temperature, turbidity, and dissolved oxygen (Supplementary Fig. 8). The strong inverse relationship between viral abundance and salinity aligns with a previous meta-analysis of 333 estimations of virus abundance in surface waters [53]. This reinforces the significant role of salinity as a key factor shaping the dynamics of viral communities in aquatic ecosystems.

### Challenging the concept of strict host–virus co-speciation

In this context, we sought to determine if (i) multiple viral genomes in one lysate corresponded to closely related viruses, and (ii) the same or closely related viral strains occurred multiple times across lysates (and thus across different host strains) from different water samples. We assessed the genetic diversity of viruses isolated from either the same (within) or different lysates (between) by analyzing the pairwise amino acid differences in the DNA polymerase B gene (*polB*) sequences from our dataset. Among 80 prasinovirus genomes, 69 (89%) encoded a single full-length *polB* gene. Surprisingly, we found no significant difference in average amino acid pairwise divergences within lysates (average 161, ~16.8%) compared to between lysates (average 183, ~19.1%) (Mann–Whitney test, *P*-value = .92; Fig. 1D). Consequently, the *polB* sequence revealed similar amino acid divergence between prasinoviruses infecting the same or different hosts. This preliminary analysis highlighted that the considerable conservation of the *polB* gene may blur viral diversity on a genome-wide scale.

To further explore prasinovirus diversity, we constructed a phylogenetic tree based on *polB* protein sequences (Supplementary Fig. 9). Although *polB* is a robust marker for assessing prasinovirus diversity in environmental water samples [54, 55], its high sequence conservation tends to underestimate the overall genomic diversity. Therefore, we employed wgANI for more precise genomic identity estimations at the nucleotide level, particularly suited for incomplete and medium-quality GVMAGs [32] (Supplementary Table 3). Integrating *polB* evolutionary information with wgANI, we defined viruses with identical *polB* sequences and a wgANI >98% as the same “genotype” (Supplementary Fig. 10). Most genotypes were unique, but 33 virus genomes shared a wgANI >98% with at least one other genome. The most prevalent genotype was identified in *Ostreococcus* lysates, obtained from the *O. tauri* strain RCC4221 collected from environments HE, HG, HJ, HL, and HO; strain RCC6881 from environment HAB; and one *O. lucimarinus* strain RCC3401 from HO (Supplementary Table 3 and Supplementary Fig. 9). We subsequently excluded substandard genomes, specifically those missing three or more of the six marker genes, with poor alignment, or comprising excessive contigs. This quality control process reduced the initial set of 80 viral genomes to 51 (64%) near-complete SCS prasinovirus genotypes for further analysis.

**Figure 2:**
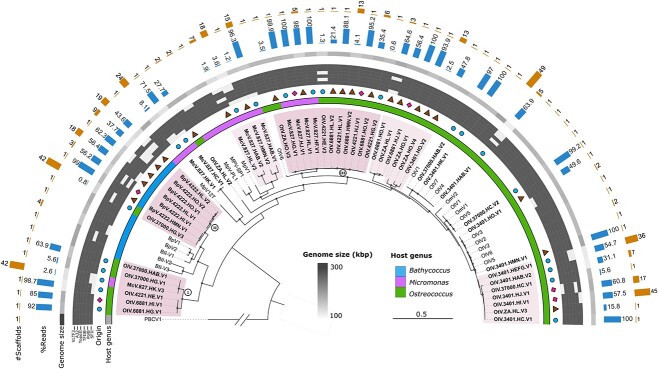
Phylogenetic diversity and assembly features of 51 virus “genotypes.” Phylogenetic reconstruction was inferred from a concatenated alignment of *SFII* (GVOGm0013), *polB* (GVOGm0054), *TFIIB* (GVOGm0172), *TopoII* (GVOGm0461), *A32* (GVOGm0760), and *VLTF3* (GVOGm0890) markers defined within the phylum Nucleocytoviricota. Twenty-two reference prasinoviruses were included with the 51 virus assemblies (3 assemblies with <3 marker genes were excluded: McV-827-HK-V4, BpV-4222-HI-V2, and OlV-3401-HJ-V2). Virus PBCV-1, which infects *P. bursaria chlorella*, was used as an outgroup and truncated for display purposes. The newly isolated prasinoviruses are shown in bold. Novel clades are shaded. Symbols indicate the geographical origin of the new virus isolates: circle (closer to open ocean: HAB-HK), diamond (intermediate: HI-HJ), and triangle (closer to the Pearl River: HM-HO). The heatmap represents the presence or absence of marker genes within genomes. The outermost circle indicates the total number of scaffolds (#scaffolds) in each genome. The next circle show the proportion of reads recruited to each assembly (%reads). The third circle represents the genome size in kilobase pairs (kbp). The three novel lineages (I, II, and III) are highlighted in red and light red. Circles mark bootstrap values >85%. The scale bar represents the number of estimated substitutions per site. The single-protein phylogenetic trees are available in the supplemental data (Figs. 8–13).

Using six conserved marker genes (*SFII*, *polB*, *TFIIB*, *TopoII*, *A32*, and *VLTF3*) from the phylum *Nucleocytoviricota* [8], we constructed a phylogenetic tree which revealed that viruses infecting the same host strains can belong to distant lineages, while phylogenetically close viruses may infect different genera (Fig. 2). Three “outlier” virus assemblies have been identified with unexpected phylogenetic positions, diverging from known *Prasinovirus* evolutionary patterns [56], and providing valuable insights into the complex virus–host interactions in marine ecosystems. The first notable case involves the *Ostreococcus*-infecting virus OlV-37 000-HG-V3, which is grouped with the prasinovirus clade typically infecting *Bathycoccus* (Fig. 2). Despite this classification, the HG water sample containing this virus failed to infect *Bathycoccus* RCC4222, a strain susceptible to other viruses within the same clade (Fig. 1B). While this observation is intriguing, it is important to note that many viruses exhibit strain specificity. Without testing OlV-37 000-HG-V3 against other *Bathycoccus* strains, we cannot conclusively determine whether this represents a host switch or simply strain-specific infection patterns. Further investigation is needed to fully understand the host range and evolutionary history of this virus. Similarly, the *Micromonas*-infecting virus, McV-827-HK-V3, phylogenetically aligned with viruses infecting *Ostreococcus* (Fig. 2). However, the HK water sample carrying McV-827-HK-V3 failed to infect the typical algal hosts (BCC37000, RCC4221, and RCC6881) of this virus clade (Fig. 1B). This pattern further supports the notion of host-switching, indicating specific adaptations to a new host. Conversely, the *Ostreococcus-*infecting virus OtV-ZA-HO-V3 is positioned within a prasinovirus clade with viruses infecting *Bathycoccus*, *Ostreococcus*, or *Micromonas* strains. This group appears to serve as a transitional clade, potentially infecting a broader array of hosts. This suggests an evolutionary adaptation that allows these viruses to extend their host range across multiple closely related species, highlighting the complex interplay of evolutionary pressures and host–virus dynamics. This unexpected phylogenetic dispersion of strains infecting the same host explains the similar amino acid divergences in the *polB* sequence observed within and between lysates (Fig. 1D). Interestingly, the three outlier viruses (OlV-37 000-HG-V3, McV-827-HK-V3, and OtV-ZA-HO-V3) occur less frequently in the lysates than other viruses, with a significant lower relative read abundance (Fig. 2, Mann–Whitney test, *P*-value = .032), suggesting lower initial frequency or lower virulence. The absence of similar genome sequences in the lysates of the same water in different strains, coupled with the absence of a double peak in the 18S rRNA sequencing chromatogram, effectively rules out algal host contamination. This reinforces the reliability of the host affiliation of these outlier viruses (Fig. 2) and support the hypothesis of host range expansion. This challenges the prevailing view that most prasinoviruses are strain specific and have co-speciated with their host [57, 58] as previously isolated members of each prasinovirus clade were observed to infect only the same species or genus. This traditional perspective may be skewed by isolating more virulent strains, and our results suggest that host-switching across genera could be more frequent than previously inferred from virus isolation efforts. Moreover, 6% of virus genomes (3 out of 51) failed to infect a host from the same genus as previously isolated despite having highly similar genomic sequences. Reciprocally, the method of inferring the host genus based on the sequence similarity of viral genomes proved accurate in 94% of cases within this model system.

### Expanded prasinovirus genome resource reveals a finite set of genes

Pan-genomic analysis revealed a set of 630 orthogroups among prasinoviruses (Supplementary Table 4). The rarefaction curves for unique genes approached saturation, indicating that our dataset has a closed pan-genome (alpha > 1) (Supplementary Fig. 10), where new additional genomes would now contribute minimally to the overall gene pool of prasinoviruses. However, expanding the host-targeted enrichment strategy to include more strains could lead to the identification of additional specific prasinovirus genes in future studies.

**Figure 3:**
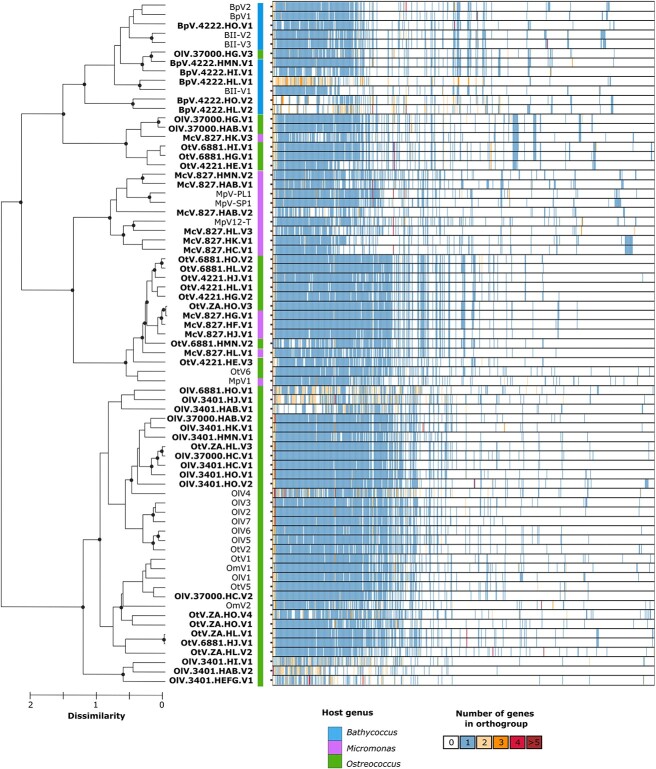
Gene family distribution within prasinoviruses. Hierarchical clustering of prasinoviruses was based on the presence/absence matrix of orthogroups. The heatmap shows the occurrence pattern of all orthogroups, ordered by frequency on the *x*-axis. Only bootstrap support values >80% are shown on the cladogram.

Further analysis based on the gene family content of the entire prasinovirus dataset revealed that the hierarchical clustering topology (Fig. 3) aligns with gene marker–based phylogeny (Fig. 2). Clade I, which is adjacent to *Bathycoccus* viruses and includes *Ostreococcus* viruses as well as the McV-827-HK-V3 virus, shares 18 unique orthogroups, predominantly of unknown function. Two genes in this clade are linked to Class-I S-adenosylmethionine (*SAM*) methyltransferases (*Mtases*). Clade II, grouping OlV-37 000-HG-V3 with *Bathycoccus* viruses, shares 24 unique orthogroups; of these, three have predicted functions: two are *SAM-Mtases* and one is a starvation-inducible transcriptional regulator protein, sharing 51% amino acid identity with a protein from Yellowstone Lake phycodnavirus 2. Mtases are epigenetic modification enzymes commonly found in giant virus genomes [1, 59]. In the *Phycodnaviridae* family, chloroviruses encode complete restriction-modification (R-M) systems where *Mtases* are associated with companion DNA site-specific (restriction) endonucleases (*REases*) [60], with a bacterial ancestry. While prokaryotic *Mtases* sometimes appear without cognate REases (dubbed orphans) and serve various biological functions [61, 62], the function of similar orphan *Mtases* in viruses remains unknown [63]. In our study, no *Mtases* were linked to *REases* companions in these specific orthogroups, suggesting potential alternative functions in prasinoviruses. Interestingly, two orthogroups (OG0000421 and OG0000422) associated with *Mtases* in the *Bathycoccus* group and OlV-37 000-HG-V3 are orthologous to the *Mtases* of the distantly related chloroviruses infecting the freshwater *Chlorella* species (Supplementary Table 4).

In Clade III, 15 predicted proteins are specific orthologs to the 13 studied viruses and are novel among prasinoviruses. Of these, only two have assigned putative functions: one is a haloacid dehydrogenase (*HAD*) family hydrolase, and the other is a collagen-like protein. Furthermore, two protein sequences resemble a cupin domain-containing protein and a FkBM *Mtases*, respectively. The cupin domain is a conserved protein fold associated with enzymatic activities, and often involved in metal binding and catalysis. FkBM *Mtases* is an enzyme that methylates specific DNA sequences, crucial for genetic regulation.

Among the three outlier viruses discussed, only OlV-37 000-HG-V3 harbors a unique gene (OlV-37 000-HG-V1-00211) encoding a hypothetical protein. This gene showed low sequence similarity (30% amino acid identity) and coverage (30%) with a bacterial sequence in the NCBI database (WP_292229369.1), underscoring the distinctiveness of this virus and suggesting a potentially novel role in viral biology or host interaction.

### Comparison of viral genome assemblies from host-targeted enrichments and metagenomic assemblies

To assess the prevalence and distribution of the new SCS viral genomes in the global viral metagenomic dataset, we screened the Global Ocean Eukaryotic Viral (GOEV) [16] for sequences matching the *polB* protein from our assemblies (>80% identity; *e*-value > 1*e*^−5^; alignment length > 500 bp). We identified 39 *Nucleocytoviricota* GVMAGs, all belonging to the *Prasinovirus* genus within the *Phycodnaviridae* family, predominantly related to *Micromonas* virus lineages. While six GVMAGs aligned more closely with novel lineages within clade I, none were associated with lineages in clades II and III (Supplementary Fig. 11), indicating potential geographical barriers or environmental influences on the diversity of giant virus genotypes in the SCS.

Using wgANI analysis [32], we compared the recovered GVMAGs with our SCS viral genomes, confirming low similarity and identifying 17 novel prasinovirus lineages in SCS coastal waters, including 5 in clade II and 11 in clade III and OtV-ZA-HL-V2 (Supplementary Figs. 12 and 13). Furthermore, our comparative analysis on genome completeness showed that our enrichment approach yielded higher completeness for SCS viral genomes than the TARA dataset (Mann–Whitney test, *P*-value < .01) and was comparable to GVMAGs from Moniruzzaman *et al*. [17] (Mann–Whitney test, *P*-value > .05) (Supplementary Table 5). However, while GVMAGs from Schulz *et al*. [16] exhibited higher completeness (Mann–Whitney test, *P*-value < .01), their results should be interpreted with caution. These prasinovirus GVMAGs often had >50 contigs and genome sizes outside the typical 170–230 kb range for isolated prasinoviruses, suggesting potential binning errors that could affect the accuracy of completeness assessments.

Our enrichment method is the only technique that successfully produced closed prasinovirus genomes, with nine complete genomes confirmed by CheckV [64]. This approach achieved a 17.6% success rate in acquiring complete genomes, significantly outperforming the 2.5% rate found in the IMG/VR 2.0 database. Unlike culture-independent methods that rely on sequence similarity, our strategy increases target DNA quantity and provides accurate host-specificity information, which is crucial for understanding host–virus interactions and the ecological impact of prasinoviruses in marine ecosystems.

### Evidence of horizontal gene transfer between hosts and viruses

**Figure 4:**
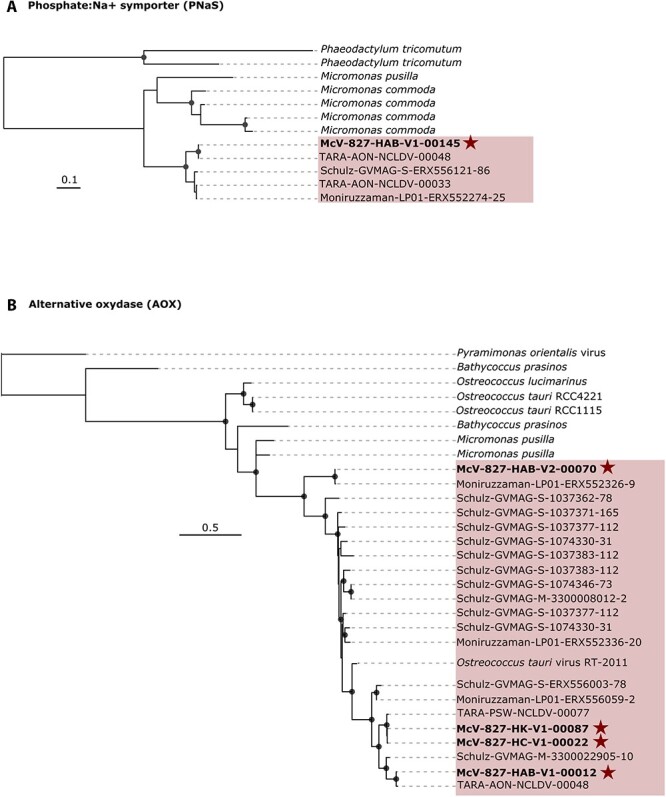
Evidence of recent lateral gene transfer between green alga host and viruses. Maximum-likelihood phylogenetic reconstruction of (A) phosphate:Na + symporter (PNas) protein and (B) alternative oxidase (AOX) protein. The virus clade is outlined in red. SCS viruses in this study are bolded and marked with a star. Circles indicate bootstrap values >85%. The scale bar represents the number of estimated substitutions per site.

Horizontal gene transfers are significant drivers of viral evolution, typically involving the incorporation of host genes during infection [65–67]. This process allows viruses to manipulate host replication and defense machinery to ensure successful replication [17, 68, 69]. Our analysis identified six viral homologues with >60% amino acid similarity to host genes, primarily associated with transporters/symporters and enzymatic metabolism (Supplementary Table 6). Notably, the alternative oxidase (*AOX*) and phosphate:Na + symporter (*PNaS*) genes were found in 27 GVMAGs in the GOEV database. Phylogenetic evidence suggests that these genes were recently acquired through lateral transfer from hosts (Fig. 4). The *AOX* gene was detected in 22 prasinoviral assemblies, including 17 GVMAGs, with 3 showing duplications, whereas the *PNaS* gene was less common, appearing in only 5 viruses (Fig. 4).

The *AOX* gene encodes an enzyme critical to the respiratory electron transport chain in plants, algae, fungi, and protists [70, 71]. It also helps in preventing reactive oxygen species (ROS) production under stress in plants [72] and algae [73]. During viral infection and replication, ROS levels can spike, causing oxidative stress that damages cellular components like DNA, proteins, and lipids. This can lead to cell death and impede successful virus replication. By acquiring genes like *AOX*, viruses might delay cell death, prolonging virus production. While giant viruses are known to possess genes like superoxide dismutase enzyme and glutathione peroxidase for managing oxidative stress [17, 74–77], the identification of *AOX* as a viral homolog in a giant virus has never been reported to our knowledge. This finding highlights the diverse adaptation strategies prasinoviruses use to mitigate oxidative stress during infection.

In addition to the essential macronutrients required for phytoplankton growth, proteins like nutrient uptake transporters can be limiting in oligotrophic environments [78, 79]. Marine viruses often possess ammonium and phosphate transporters [69, 80, 81], with inorganic phosphate (Pi) transporters previously documented in giant viruses [16, 17, 80, 81]. However, the *PNaS* gene is rare in the marine environment [17]. Unlike the PO_4_ transporter that operates on a phosphate ion concentration gradient, *PNaS* utilizes the energy from the electrochemical gradient of sodium ions (Na^+^) to actively transport Pi against its gradient. This may allow simultaneous uptake of Na^+^ and Pi into virus-infected cell. Our data show that only one *Micromonas* virus (McV-827-HAB-V1-00145) and *Ostreococcus* virus OtV6 encode *PNaS* genes. The scarcity of *PNaS* genes among marine viruses suggests that this transporter may have evolved to meet specialized environmental needs. The strategic use of these transporters by viruses likely enhances their ability to manipulate host nutrient uptake, improving their replication and survival. Nevertheless, further research is needed to explore how viral infection, nutrient limitation, and transport mechanisms interact, to deepen our understanding of virus–host eco-evolutionary dynamics.

### Diversifying selection on amino acid evolution in the virus capsid proteins

Diversifying selection is a cornerstone of the co-evolutionary arms race between hosts and viruses, yet its documentation in giant viruses is limited, likely because of the lack of appropriate available datasets. We applied a maximum-likelihood approach to detect signatures of diversifying (or positive) molecular evolution [47, 82] in the Major Capsid Protein (*MCP6*) gene by analyzing the ratio of non-synonymous (*dN*) to synonymous (*dS*) substitutions (*ω*). To ensure accurate estimations, we partitioned the global alignment into 29 sub-alignments to avoid saturation at synonymous sites, to enhance the accuracy of the *dN*/*dS* ratio (*ω*) estimation.

We compared the diversifying selection model (M2a), which allows for a proportion of sites with *ω* >1, against the purifying selection model (M1a), which includes sites with *ω* <1 and neutral evolution (*ω* = 1), across seven datasets (Table 1 and Supplementary Table 7). The M2a model consistently outperformed the M1a model, suggesting a better fit and confirming diversifying or positive selection acting on specific amino acids within the *MCP6* gene. Moreover, Bayes empirical Bayes analysis [83] identified 1 to 31 amino acid sites under diversifying selection across different datasets. These findings support previous evidence that diversifying selection impacts viral capsid proteins, which are involved in physical interactions with the host [84].

**Table 1:** Likelihood values and parameter estimates for major capsid protein alignments with evidence for diversifying selection.

**Alignment**	***n***	***l***_***M1***_	***l***_***M2***_	**2*Δl***	***P-value***	*p_2_*	***ω**_2_*	**BEB sites with** ***ω*** **> 1**
m0003-G2-02-a	5	−2538.7	−2507.8	61.8	<.0001	.19	102.6	34 sites^[1]^
m0003-G2-02-b	6	−2161.3	−2153.7	15.1	<.0001	.21	9.9	6 sites^[2]^
m0003-G2-04-b	11	−3650.1	−3647.0	6.2	<.05	.006	11.1	212 (N)
m0003-G2-05-a	7	−3585.0	−3578.2	13.6	<.001	.08	8.9	243 (W), 300 (Q)
m0003-G2-05-b	5	−3635.7	−3631.5	8.2	<.05	.07	31.4	
m0003-G3-01	3	−2472.3	−2465.4	13.8	<.001	.12	111.8	9 sites^[3]^
m0003-G4-03	6	−1357.4	−1353.3	8.3	<.05	.06	5.1	29 (S) 75 (Q)

[1] 74 (P), 79 (K), 82 (K), 86 (S), 101 (T), 106 (Q), 107 (Y), 108 (I), 113 (L), 114 (A), 116 (N), 117 (L), 118 (T), 120 (S), 122 (S), 123 (G), 124 (F), 160 (I), 162 (S), 163 (E), 218 (A), 224 (M), 235 (F), 237 (Y), 239 (D), 244 (A), 247 (S), 252 (P), 254 (S), 256 (D), 257 (E), 261 (F), 263 (Y), 294 (N)

[2] 130 (T), 133 (W), 190 (T), 250 (T), 278 (M), 310 (S)

[3] 87 (D), 151 (P), 158 (L), 223 (E), 224 (S), 266 (P), 268 (N), 300 (F), 301 (V)

*n*, number of sequences in alignment; *l*, loglikelihood of dataset under model M1 or M2; *p_2_*, proportion of sites with *ω_2_* >1; *ω_2_*, positive selection coefficient; BEB, Bayes empirical Bayes analysis of positively selected sites with Pr(*ω_2_* > 1) >.95, position (amino acid).

**Figure 5:**
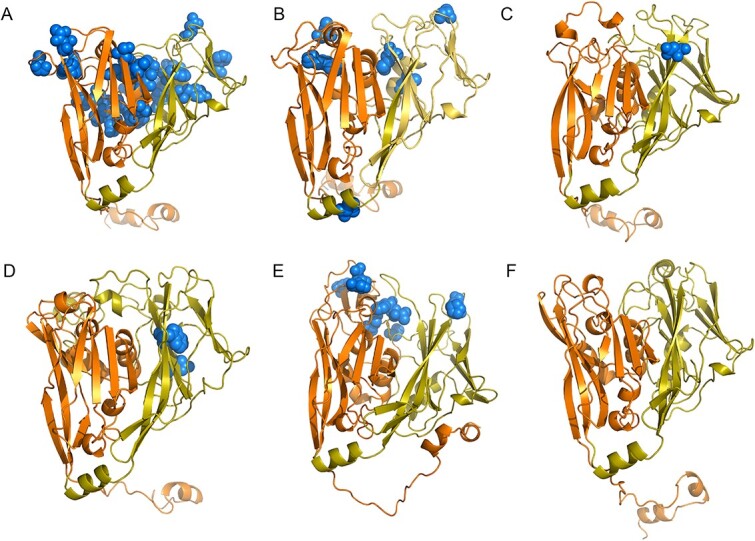
Evidence of diversifying selection detected in the major capsid protein (MCP) among green algal viruses. Predicted three-dimensional (3D) structures of the MCP in the alignments are shown for (A) m0003-G2-02-a, (B) m0003-G2-02-b, (C) m0003-G2-04-b, (D) m0003-G2-05-a, and (E) m0003-G3-01. (F) *Paramecium bursaria* chlorella virus 1 (PBCV-1, accession: NP_048358). Domains D1 and D2 are indicated in orange and yellow, respectively. Spheres represent the residues under selection based on PAML analysis. The 3D structures of m0003-G4-03 and m0003-G2-05-b (Table 1) are not included in this visualization due to unsuccessful predictions by AlphaFold.

To elucidate the structural implications of the amino acid sites under diversifying selection, we employed AlphaFold v2.0 [85] to predict the three-dimensional conformation of the *MCP6* protein. This analysis showed notable structural resemblance between the predicted *MCP6* capsid structure and that of *Paramecium bursaria* Chlorella virus 1 (PBCV-1) [86] (Fig. 5, Supplementary Table 8), despite only 25% amino acid identity between the chlorovirus and prasinovirus proteins. Both structures exhibit two jelly-roll domains, a common motif in viral capsid proteins characterized by four antiparallel beta strands in a β-meander configuration [87]. This similarity underscores the functional importance and conservation of jelly-roll domains in capsid proteins.

Interestingly, the sites undergoing diversifying selection within MCP6 are primarily located on one facet of the capsid protein, predominantly on the exterior surface. This spatial distribution is as expected because external sites are more likely to be involved in environmental interactions, possibly with host cell receptors. Previous research supports the idea that surface-exposed regions of capsid proteins play critical roles in virus–host interactions, including host recognition, receptor binding, and immune evasion strategies [88]. The presence of diversifying selection on these surface-exposed regions further emphasizes their functional importance in the interactions between the virus and its host.

## Conclusions

Targeted virus enrichment led us to sequence 51 new prasinovirus viral genomes associated with known algal host from the SCS. Comparative analyses with 22 prasinovirus reference genomes and available metagenome-assembled giant virus genomes allowed us to track their evolutionary trajectories. We identified a distinct prasinovirus subgroup in SCS coastal waters with 15 additional orthogroups, suggesting that environmental factors or ecological interactions might have shaped their genome evolution. Our findings challenge the notion of strict host–virus co-speciation and reveal substantial genomic diversity within the same lysate.

We identified six genes transferred from Mamiellophyceae hosts to prasinoviruses, including *AOX* and *PNaS*. *AOX* potentially prolongs viral replication by delaying host apoptosis, while *PNaS* may enable viruses to manipulate host nutrient uptake in oligotrophic environments. These findings, significant beyond the SCS, highlight the prevalence of specific gene families acquired *via* HGT from host genomes. Additionally, our analysis of the *MCP6* gene indicates adaptive evolution with diversifying selection on sites likely critical for host–virus interactions, predominantly affecting the protein’s external surface, which could influence interactions with host cellular receptors.

Overall, this study not only deepens our comprehension of prasinovirus diversity in the SCS but also underscores the significance of viral dynamics in marine ecosystems. By exploring evolutionary strategies like HGT and diversifying selection, our research sets the stage for further investigations into the ecological and evolutionary impacts of these genomic adaptations. Our findings illuminate the complex interactions between viruses and their hosts, underscoring the need for expanded experimental and functional studies. By producing lysates for further experiments, our host-targeted enrichment approaches facilitate forthcoming investigations into the intricate virus–phytoplankton interplay.

## Supplementary Material

Supplementary_figures_submission_final_ycae109

Supplementary_tables_final_ycae109

## Data Availability

All genomic sequences of prasinovirus enrichment cultures from the South China Sea targeting Mamiellales green algae have been submitted to GenBank under Bioproject number PRJNA1092634 (https://dataview.ncbi.nlm.nih.gov/object/PRJNA1092634?reviewer=26bcfgbuff6aa0vtfdo54c5l1s).
