# Supplementary material for: Unveiling Prasinovirus diversity and host specificity through targeted enrichment in the South China Sea
Source: ISME Commun. 2024 Aug 29;4(1):ycae109. doi: 10.1093/ismeco/ycae109 (PMC11408933; doi:10.1093/ismeco/ycae109)
Supplement: Supplementary_figures_submission_final_ycae109 [file supplementary_figures_submission_final_ycae109.docx]

Figure S1. Host targeted viral enrichment sequencing pipeline


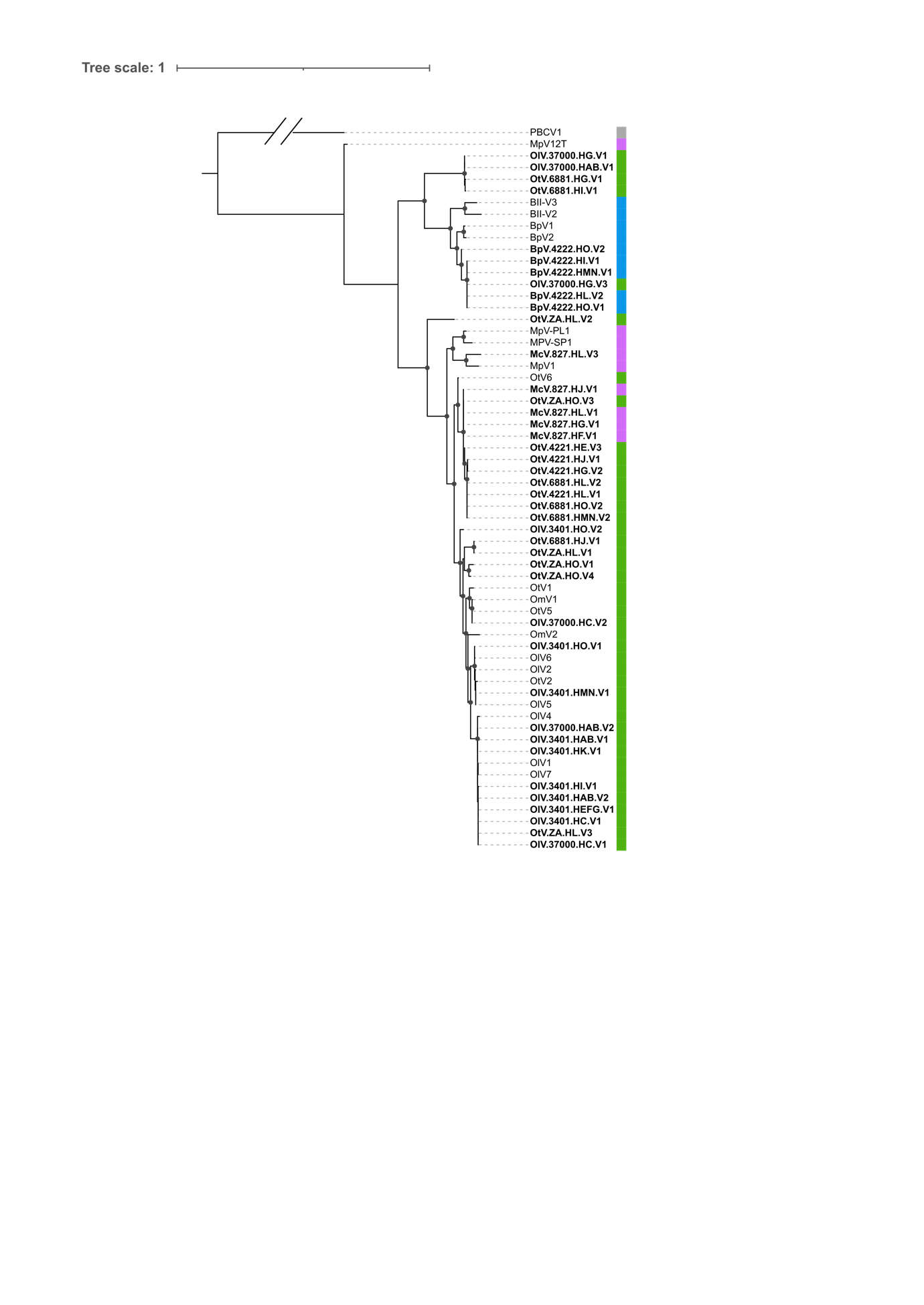


Figure S2. Phylogenetic tree of DEAD/SNF2-like helicase protein (SFII - GVOGm0013) from the 41 new and 20 reference prasinoviruses. The tree was reconstructed using the best Bayesian inference criterion (BIC), the LG+R3 model. The chlorovirus PBCV-1 was used as the outgroup, with the connecting branch truncated for display purposes. Colours represent the host genus that they infect: blue for *Bathycoccus*, purple for *Micromonas*, and green for *Ostreococcus*. Newly isolated viruses in this work are highlighted in bold. Bootstrap values with support higher than 80% are indicated on the branches. The scale shows substitutions per site.


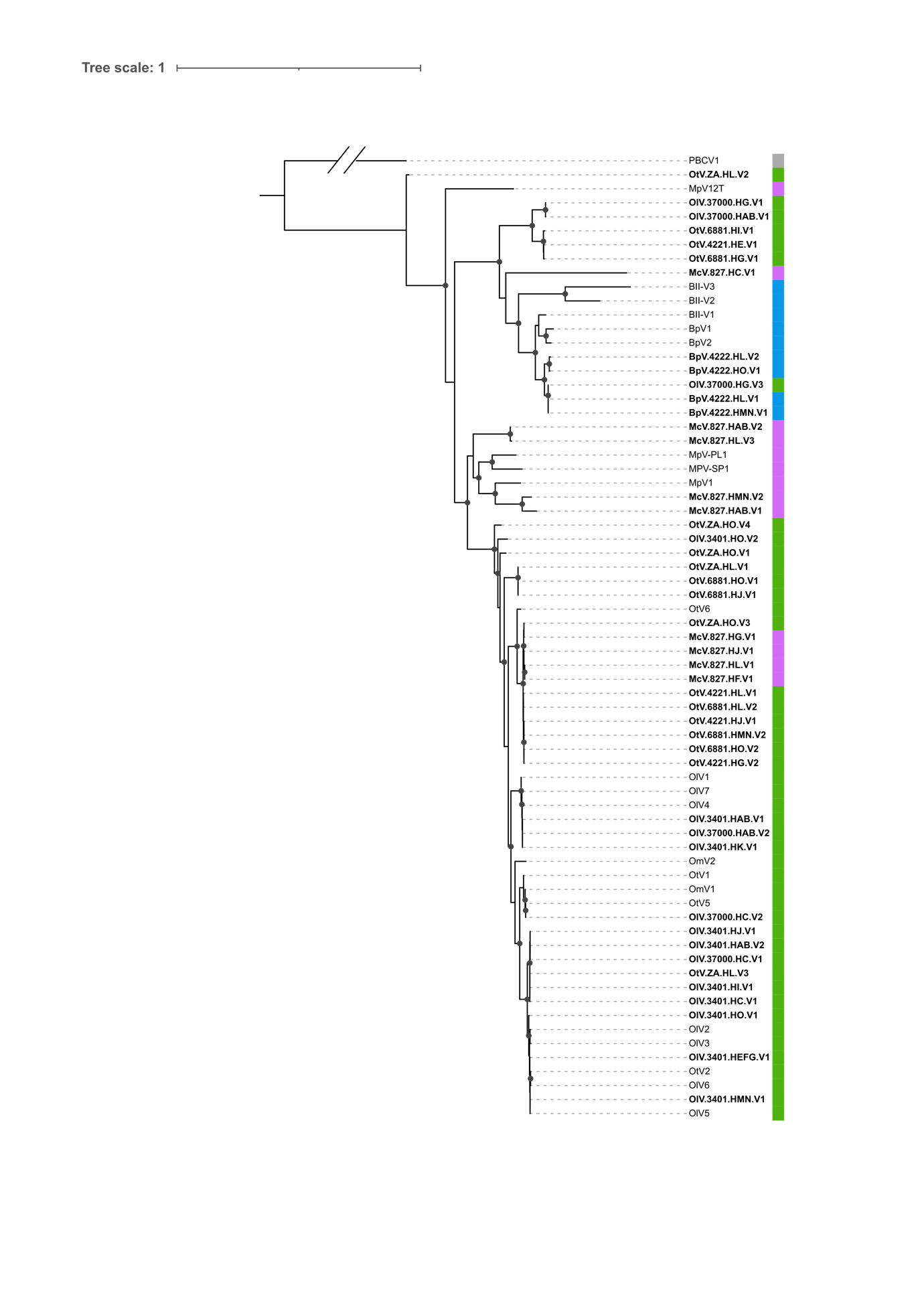


Figure S3. Phylogenetic tree of DNA polymerase family B protein (PolB - GVOGm0054) from the 46 new and 22 reference prasinoviruses. The tree was reconstructed using the best Bayesian inference criterion (BIC), the LG+R4 model. The chlorovirus PBCV-1 was used as the outgroup, with the connecting branch truncated for display purposes. Colours represent the host genus that they infect: blue for *Bathycoccus*, purple for *Micromonas*, and green for *Ostreococcus*. Newly isolated viruses in this work are highlighted in bold. Bootstrap values with support higher than 80% are indicated on the branches. The scale shows substitutions per site.


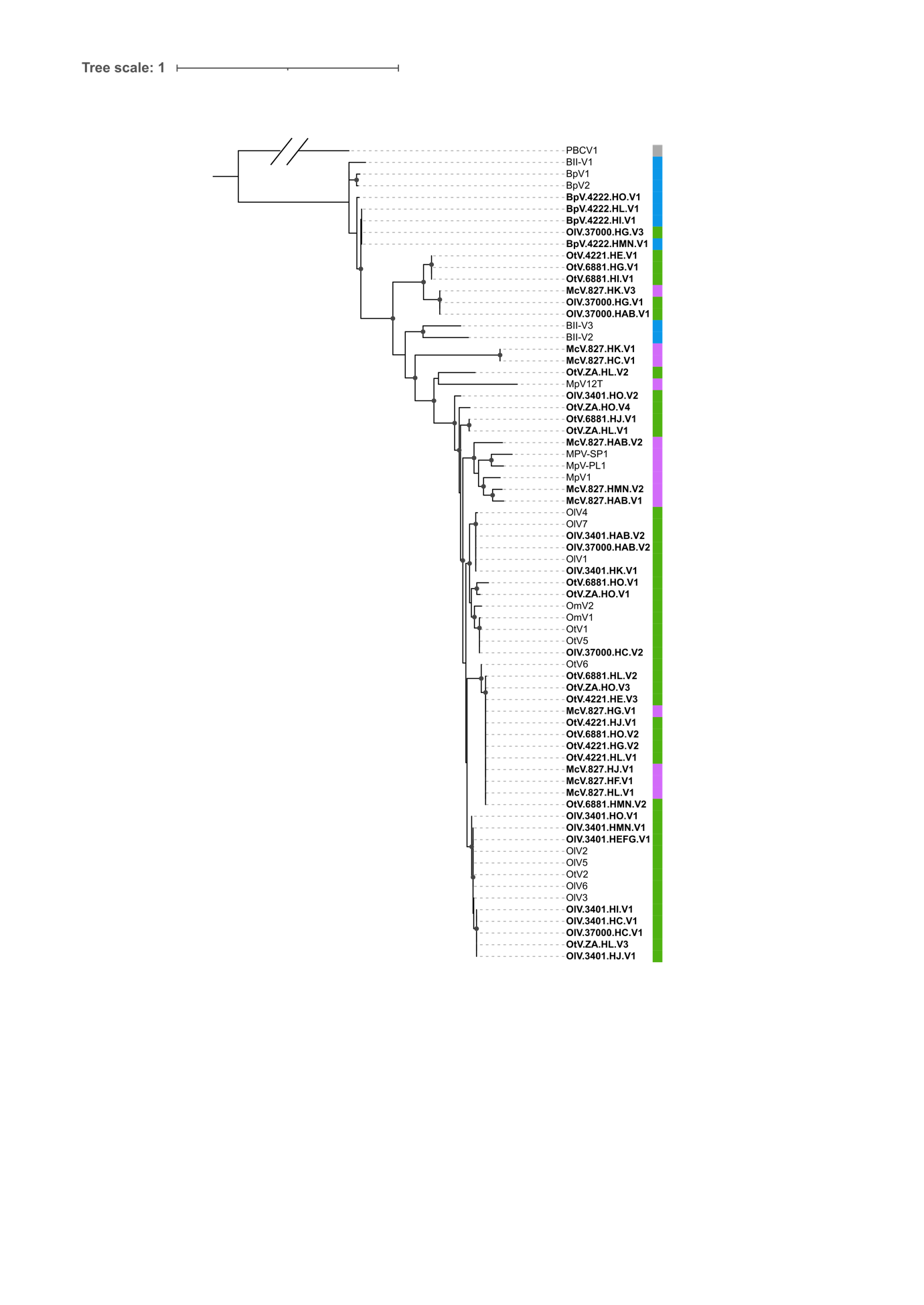


Figure S4. Phylogenetic tree of Transcription initiation factor IIB protein (TFIIB - GVOGm0172) from the 47 new and 22 reference prasinoviruses. The tree was reconstructed using the best Bayesian inference criterion (BIC), the LG+R3 model. The chlorovirus PBCV-1 was used as the outgroup, with the connecting branch truncated for display purposes. Colours represent the host genus that they infect: blue for *Bathycoccus*, purple for *Micromonas*, and green for *Ostreococcus*. Newly isolated viruses in this work are highlighted in bold. Bootstrap values with support higher than 80% are indicated on the branches. The scale shows substitutions per site.


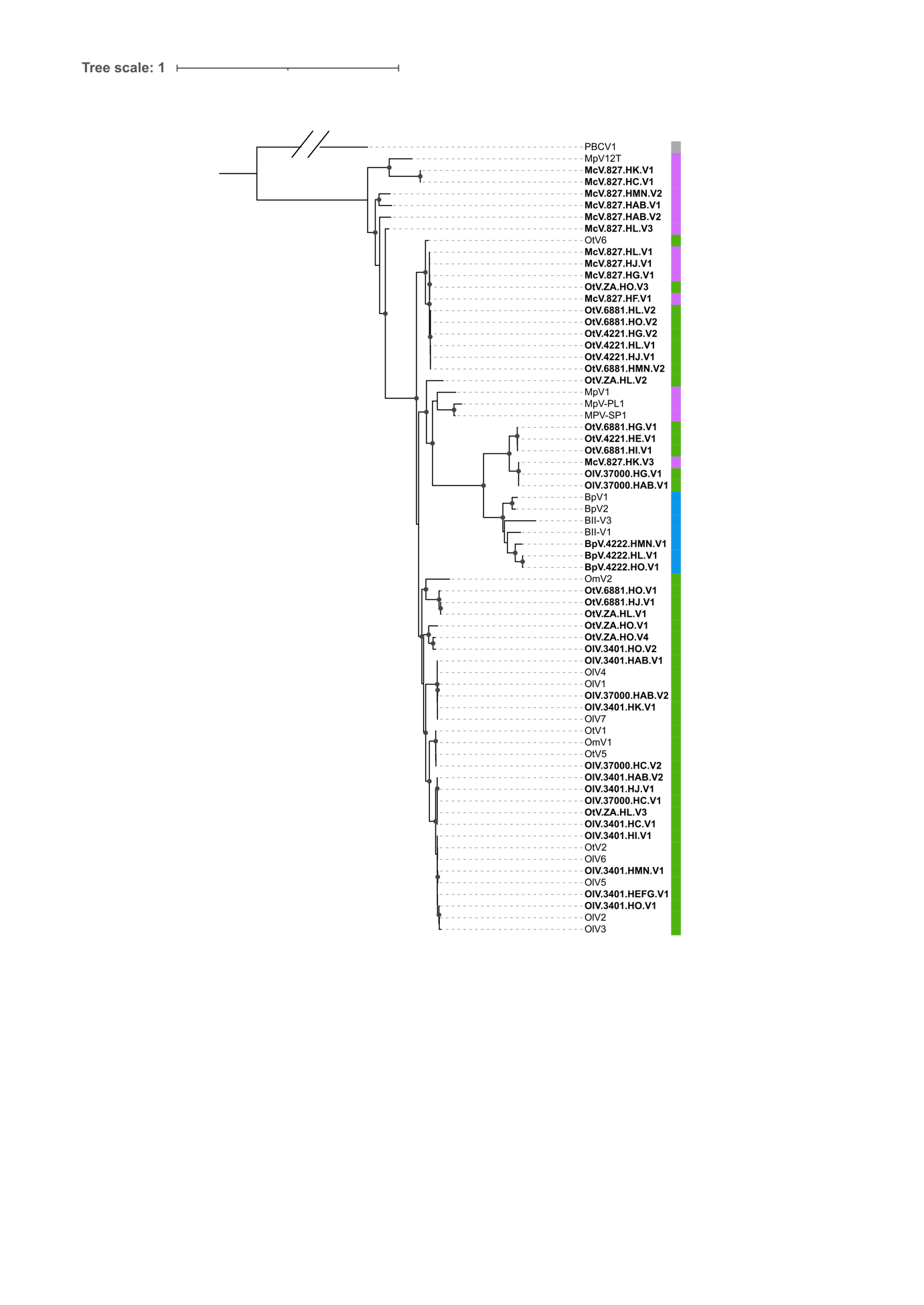


Figure S5. Phylogenetic tree of DNA topoisomerase II protein (TopoII - GVOGm0461) from the 46 new and 21 reference prasinoviruses. The tree was reconstructed using the best Bayesian inference criterion (BIC), the LG+R3 model. The chlorovirus PBCV-1 was used as the outgroup, with the connecting branch truncated for display purposes. Colours represent the host genus that they infect: blue for *Bathycoccus*, purple for *Micromonas*, and green for *Ostreococcus*. Newly isolated viruses in this work are highlighted in bold. Bootstrap values with support higher than 80% are indicated on the branches. The scale shows substitutions per site.


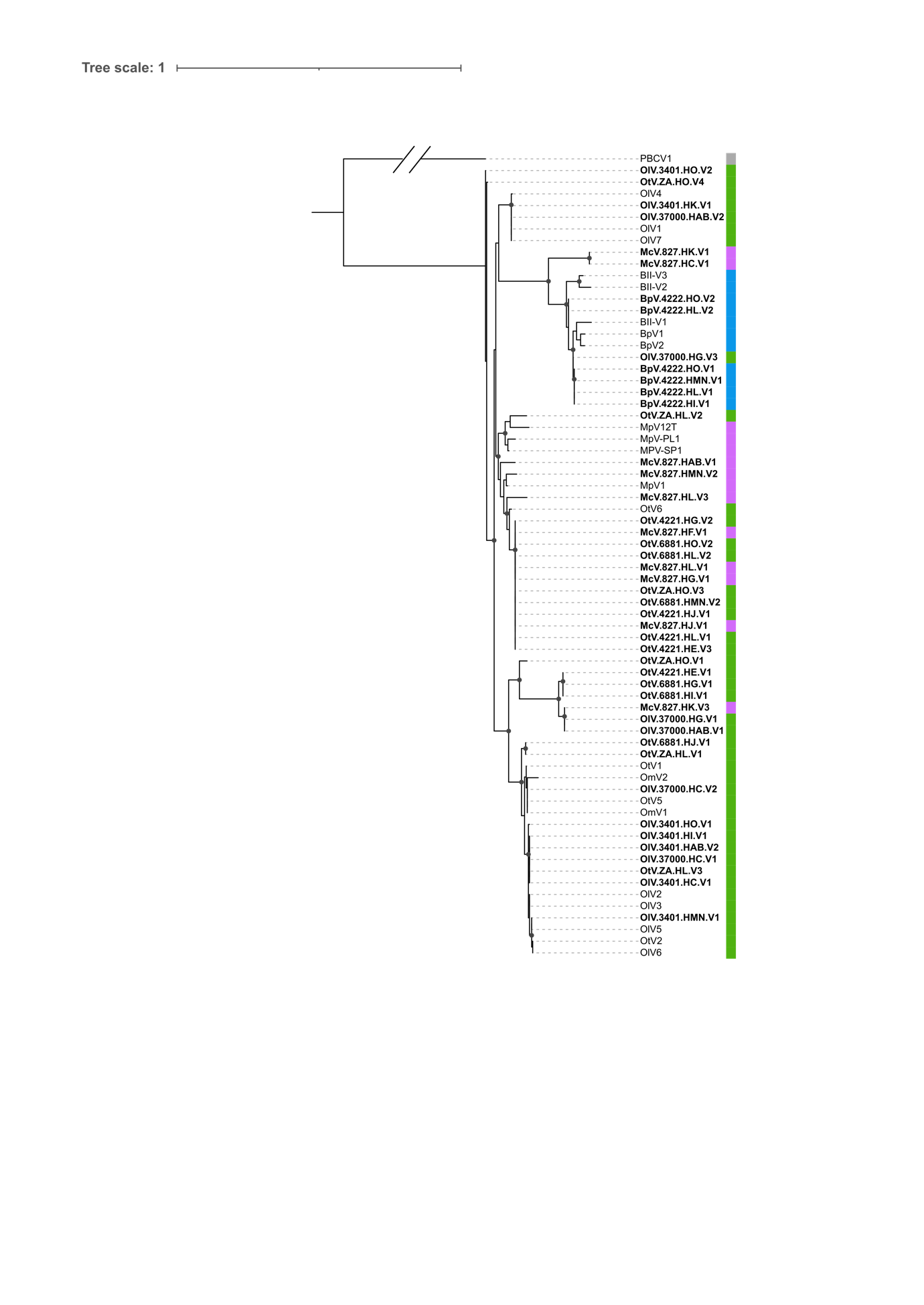


Figure S6. Phylogenetic tree of Packaging ATPase protein (A32 - GVOGm0760) from the 46 new and 22 reference prasinoviruses. The tree was reconstructed using the best Bayesian inference criterion (BIC), the LG+R3 model. The chlorovirus PBCV-1 was used as the outgroup, with the connecting branch truncated for display purposes. Colours represent the host genus that they infect: blue for *Bathycoccus*, purple for *Micromonas*, and green for *Ostreococcus*. Newly isolated viruses in this work are highlighted in bold. Bootstrap values with support higher than 80% are indicated on the branches. The scale shows substitutions per site.


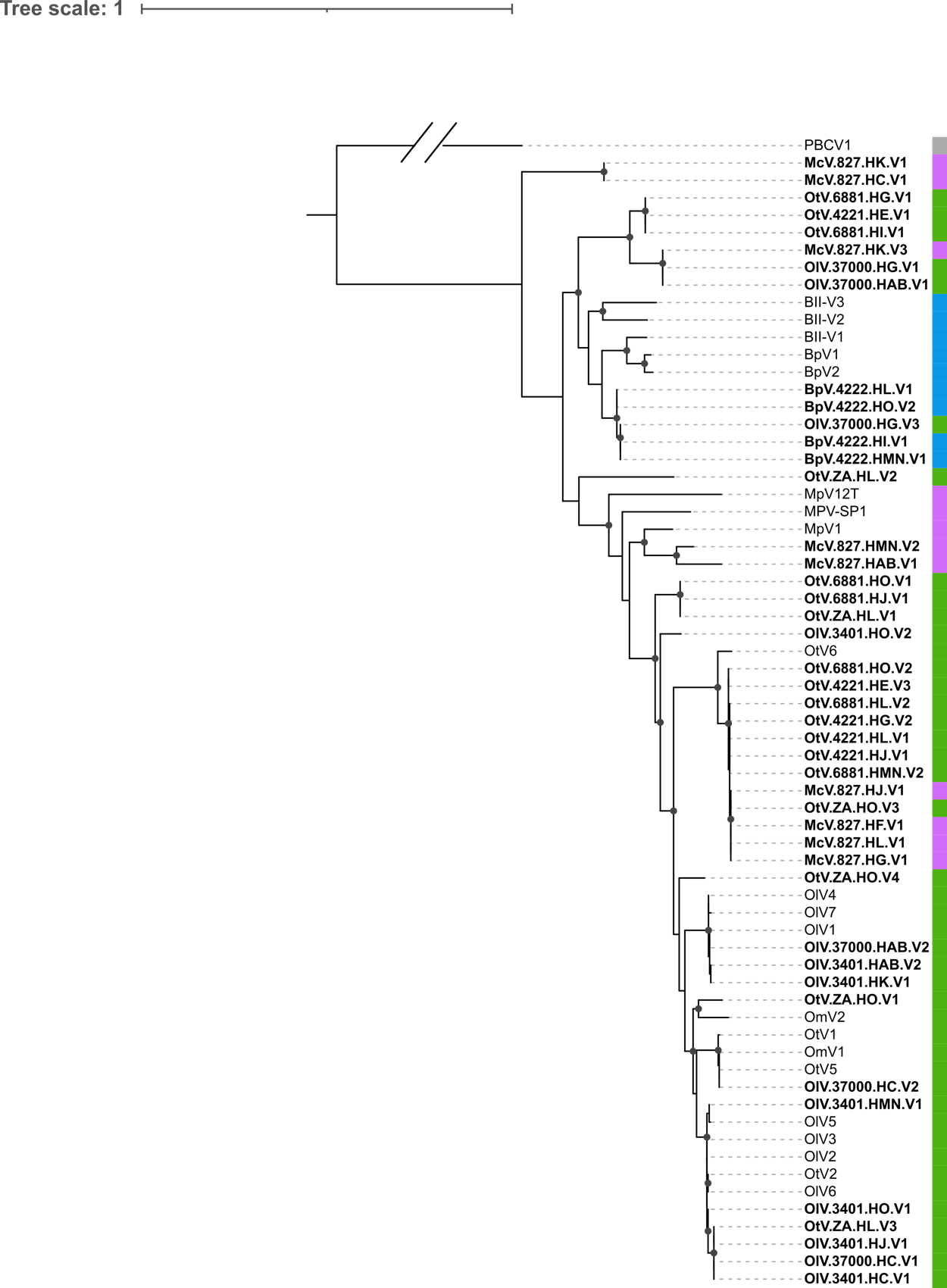


Figure S7. Phylogenetic tree of Poxvirus Late Transcription Factor VLTF3 protein (VLTF3 - GVOGm0890) from the 44 new and 21 reference prasinoviruses. The tree was reconstructed using the best Bayesian inference criterion (BIC), the LG+R4 model. The chlorovirus PBCV-1 was used as the outgroup, with the connecting branch truncated for display purposes. Colours represent the host genus that they infect: blue for *Bathycoccus*, purple for *Micromonas*, and green for *Ostreococcus*. Newly isolated viruses in this work are highlighted in bold. Bootstrap values with support higher than 80% are indicated on the branches. The scale shows substitutions per site.


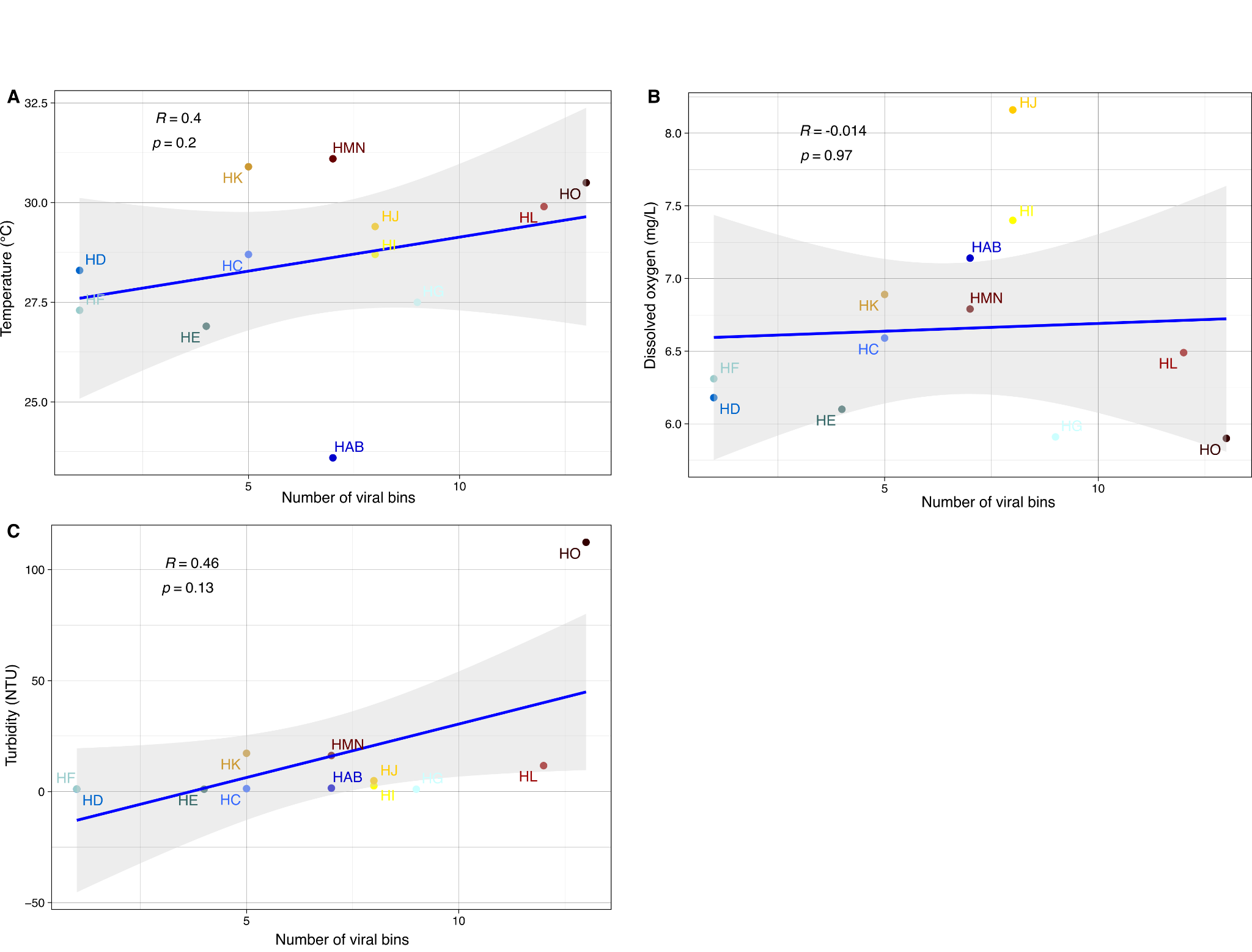


Figure S8. Scatter plot illustrating the correlation between the abundance of viral genomes and (a) temperature, (b) dissolved oxygen or (c) turbidity across 13 water samples. The 95% confidence interval is indicated in light grey. *R*: Spearman correlation coefficient. *P*: *p*-*value*.


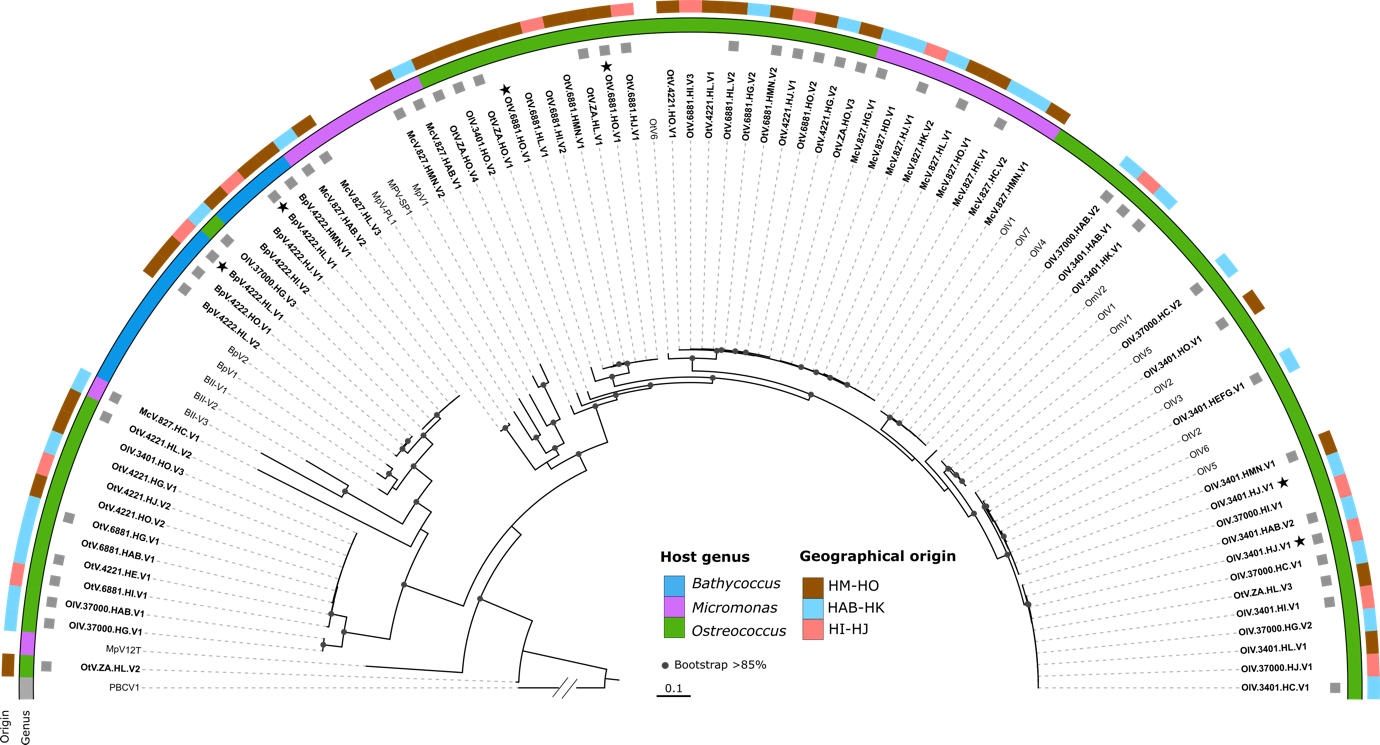


Figure S9. Maximum Likelihood phylogenetic tree of the 95 full-length DNA polymerase protein B (polB) (alignment of 957 amino-acid sites) from 80 prasinoviruses newly isolated from SCS and 22 reference prasinovirus genomes. The phylogenetic tree was constructed using the best-fit LG+R4 model. Chlorovirus PBCV-1 was used as an outgroup, with the connecting branch truncated for display purposes. The inner ring shows the host genera that they infect: blue for *Batyhcoccus*, purple for *Micromonas* and green for *Ostreococcus*. Outermost ring indicates the geographical origin of the new virus isolates: blue (closer to open ocean: HAB-HK), pink (intermediate: HI-HJ), and brown (closer to the Pearl River: HM-HO). Newly isolated viruses in this study are highlighted in bold. The 46 viruses with high genome completeness were kept for further genomic analysis (grey square). Bootstrap values higher than 85% are shown on the branches. The scale indicates substitutions per site. Viral genomes encoding two distinct polB genes are marked by a black star.

wgANI

Figure S10. Heatmap of the weighted average nucleotide identity (wgANI) calculations performed using SKANI. The arrangement of this heatmap reflects a hierarchical clustering algorithm that categorises the data based on their wgANI similarities.

Figure S11. (a) Rarefaction curves for 'pan' and 'core' genes of 73 prasinovirus genomes. The curves were fitted to the median values of 1,000 iterations following the Power Law. (b) Distribution of the genes across the 73 genomes of the prasinovirus dataset. (c-e) Rarefaction curves for 'pan' and 'core' genes of *Ostreococcus* viruses, *Micromonas* viruses, and Bathycoccus viruses, respectively. (f-h) Distribution of the genes across the genomes of *Ostreococcus* viruses, *Micromonas* viruses, and *Bathycoccus* viruses, respectively. (i) A cumulative gene accumulation graph showing the increase in gene numbers as new prasinovirus genomes are added.


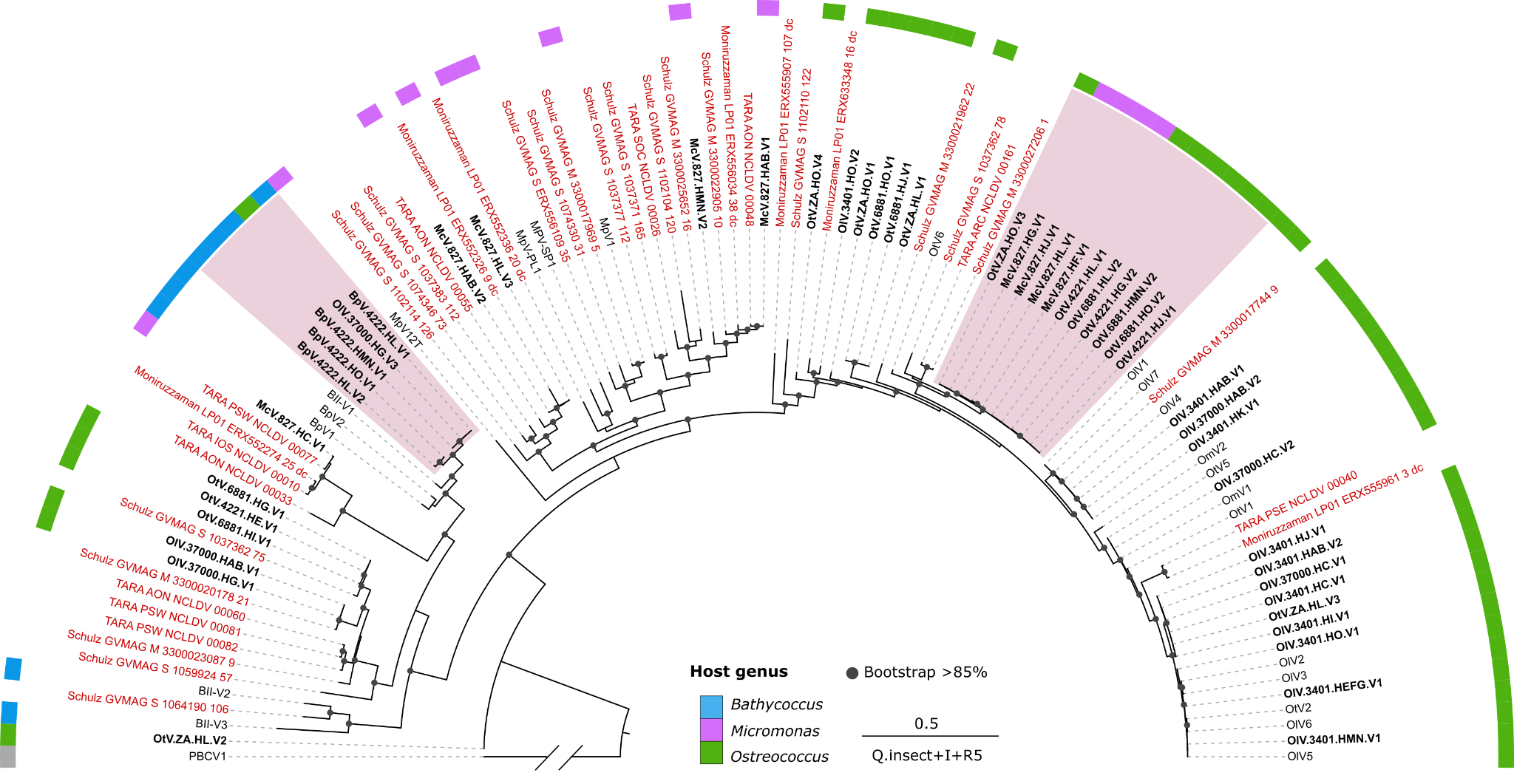


Figure S12. Phylogenetic evolutionary history of the 39 GVMAGs closely related to the 46 new viruses isolated from the SCS. PBCV-1 is used as the outgroup, with the connecting branch truncated for display purposes. The phylogenetic tree was constructed based on the Bayesian Information Criterion according to the best-fit Q.insect+I+R5 model. Viruses recovered from the metagenomics dataset in the GOEV database are shown in red, while viruses isolated in this study are highlighted in bold black. Novel clades with no previous reports and for which no viral metagenomes (GVMAGs) have been associated are also highlighted in red. Strip colours indicate the host genus that the viruses infect: blue for *Bathycoccus*, purple for *Micromonas*, and green for *Ostreococcus*. Newly isolated viruses in this study are in bold. Bootstrap values with support higher than 85% are indicated on the branches. The scale shows substitutions per site.

wgANI

Figure S13. Heatmap of the weighted average nucleotide identity (wgANI) calculations for the recovered MAGs and our SCS viral genomes, performed using SKANI. The arrangement of this heatmap reflects a hierarchical clustering algorithm that categorises the data according to their wgANI similarities.
